# Interleukin-6 is associated with tryptophan metabolism and signs of depression in individuals with carbohydrate malabsorption

**DOI:** 10.17179/excli2020-2940

**Published:** 2020-10-28

**Authors:** Dietmar Enko, Sieglinde Zelzer, Julian Wenninger, Sandra Holasek, Wolfgang J. Schnedl, Andreas Baranyi, Markus Herrmann, Andreas Meinitzer

**Affiliations:** 1 Clinical Institute of Medical and Chemical Laboratory Diagnostics, Medical University of Graz, Graz, Austria; 2 Institute of Clinical Chemistry and Laboratory Medicine, General Hospital Hochsteiermark, Leoben, Austria; 3 Department of Immunology and Pathophysiology, Medical University of Graz, Graz, Austria; 4 Practice for General Internal Medicine, Bruck/Mur, Austria; 5 Department of Psychiatry and Psychotherapeutic Medicine, Medical University of Graz, Graz, Austria

**Keywords:** depression, inflammation, interleukin-6, interferon-gamma, tumor necrosis factor-alpha, lactoferrin

## Abstract

The aim of the present study was to investigate possible associations between interleukin-6 (IL-6), interferon-gamma (INF-γ), tumor necrosis factor-alpha (TNF-α), lactoferrin and lipopolysaccharide binding protein (LBP) with TRP metabolism and signs of depression in a large cohort of outpatients referred for carbohydrate malabsorption testing. Serum concentrations of IL-6, INF-γ, TNF-α, lactoferrin, LBP, tryptophan (TRP), kynurenine (KYN) and kynuric acid were determined in 250 adults referred for lactose and fructose malabsorption testing. All participants filled out the Beck Depression Inventory (BDI). Serum IL-6 levels were positively correlated with the BDI score (p = 0.001, ρ = 0.205) and indicators of TRP metabolism (KYN/TRP ratio, KYN) (P-values < 0.05, ρ = 0.176 and 0.136). Ninety-five individuals with a BDI score > 13 showed significantly higher IL-6 serum levels (1.7 [1.0 - 2.8] vs. 1.1 [0.8 - 1.7] pg/mL, p < 0.001) compared to 115 individuals with a BDI score ≤ 13. LBP showed a positive correlation with the KYN/TRP ratio (p = 0.005, ρ = 0.177). IL-6 and LBP were associated with indicators of TRP metabolism. IL-6 was found to be linked to signs of depression. Individuals with the presence of depressive symptoms showed higher serum IL-6 levels compared to individuals without depressive symptoms.

## Introduction

Carbohydrate malabsorption is a widespread condition, which is caused by incomplete absorption of the disaccharide lactose or monosaccharide fructose. Clinical symptoms include abdominal cramps and pain, bloating, flatulence and diarrhea. Moreover, individuals with lactose and fructose malabsorption are reported to show signs of depression (Ledochowski et al., 1998[15]; Enko et al., 2018[6]).

Several recent studies provide considerable evidence, that inflammation plays a pathophysiological role in the etiology of depression (Albeltagy et al., 2020[1]; Huang et al., 2019[11]; Vogelzangs et al., 2016[29]). Pro-inflammatory cytokines, such as interleukin-6 (IL-6), interferon-gamma (INF-γ) and tumor necrosis factor-alpha (TNF-α), are elevated in depressed persons (Albeltagy et al., 2020[1]; Huang et al., 2019[11]; Postal et al., 2016[24]; Xiong et al., 2015[34]). The exact causal nature of these positive associations between markers of inflammation and depression has not been fully delineated yet.

One of the suggested mechanisms linking inflammation and depression is the cytokine-induced enzyme indoleamine 2,3-dioxygenase (IDO), which converts tryptophan (TRP) into kynurenine (KYN). Differences in the activation of IDO resulting in an increased TRP and serotonin degradation might play a key role in the pathogenesis of depression. The overweight of the pro-inflammatory cytokines IL-6, INF-γ and TNF-α in individuals with depression is associated with increased activity of IDO (Schwieler et al., 2015[25]; Müller et al., 2011[19]). Also, lipopolysaccharides (LPS) are reported inducing the IDO expression (Jung et al., 2007[13]). In clinical practice, the tryptophan break-down index (KYN/TRP) is used to assess the IDO activity (Myint et al., 2007[20]). 

Cytokine-induced IDO activation via IL-6, INF-γ and TNF-α plays an important role in immune-inflammatory dysregulation involved in depression (Barreto et al., 2018[3]). The anti-inflammatory glycoprotein lactoferrin, which is responsible for inflammatory homeostasis, regulates the release of IL-6 and TNF-α in vivo and may attenuate pro-inflammatory responses (Machnicki et al., 1993[17]; Wisgrill et al., 2018[33]). Nevertheless, human studies assessing the relationship between serum levels of pro-inflammatory cytokines (i.e., IL-6, INF-γ, TNF-α) and the anti-inflammatory lactoferrin with indicators of TRP metabolism in a large group of individuals with signs of depression are still lacking.

The aim of the present study was to investigate possible associations between the inflammatory markers IL-6, INF-γ, TNF-α, lactoferrin, and lipopolysaccharide-binding protein (LBP) with TRP metabolism and signs of depression in a large cohort of adult outpatients referred for carbohydrate malabsorption testing.

## Materials and Methods

### Study population

We investigated 250 consecutive ambulatory adult individuals, who were referred by general practitioners and specialists for carbohydrate malabsorption testing. Detailed information about the primary study has been published before (Enko et al., 2018[6]). All study participants underwent assessment of lactose and fructose malabsorption, inflammatory markers (i.e., IL-6, INF-γ, TNF-α, lactoferrin, LBP, C-reactive protein CRP), tryptophan metabolism (i.e., TRP, KYN, kynurenic acid [KYNA], tryptophan break-down index [KYN/TRP]), and renal function (i.e., creatinine, estimated glomerular filtration rate [eGFR]). Additionally, all subjects were asked to fill out the Beck Depression Inventory (BDI) questionnaire (Steer et al., 1999[27]). The inclusion criteria for this study were outpatients referred for carbohydrate malabsorption testing, a minimum age of 18 years, and an obligatory overnight fasting and non-smoking period of > 12 hours. 

### Laboratory methods

Carbohydrate malabsorption testing (i.e., lactose, fructose) was performed with standard procedures as described elsewhere (Enko et al., 2018[6]). Fasting venous blood samples were collected from 08:00 to 10:00 am. Serum IL-6 concentrations were determined with the high sensitivity IL-6 enzyme-linked immunosorbent assay (ELISA) kit from Diaclone (Besancon, France). The expected range was 0 - 4.7 pg/mL. The calculated intra- and inter-day coefficient of variations (CVs) ranged between 1.4 - 11.0 % and 4.8 - 12.2 %. INF-γ and TNF-α serum concentrations were measured with the high sensitivity INF-γ and TNF-α kits from BioVendor (Brno, Czech Republic). The expected ranges were 0.7 - 5.8 and not detectable - 3.2 pg/mL for INF-γ and TNF-α. The overall calculated intra- and inter-assay CVs were 3.9 and 8.6 % for INF-γ, and 8.5 and 9.8 % for TNF-α. Serum lactoferrin measurements were performed with the lactoferrin ELISA kit from BioVendor (Brno, Czech Republic). The expected range was 95 - 1368 ng/mL. The intra- and inter-assay CVs varied between 3.2 - 3.4 and 4.5 - 6.2 %. The LBP and CRP were measured by chemiluminescent technology and by nephelometry on a Dimension Vista^® ^1500 System (Siemens Healthineers, Erlangen, Germany).

Indicators of tryptophan metabolism (i.e., TRP, KYN, KYNA) were measured by high-performance liquid chromatography with a simultaneous ultraviolet and fluorometric detection system (Hervé et al., 1996[10]). In brief, 100 µL plasma sample was deproteinized by adding 100 µL of 5 % (v/v) perchloric acid. After vortexing and 5 min centrifugation at 11,000 x g, 20 μL of the clear supernatant was injected into the chromatographic system. Separations were achieved on a Chromolith RP18e column (100 x 4.6 mm, 5µm, Merck Darmstadt, Germany) at 30 °C by isocratic elution with a mobile phase (pH 4.9) consisting of 50 mmol/L ammonium acetate, 250 mol/L zinc acetate and 3 % (v/v) acetonitrile, at a flow-rate of 0.8 mL/min. TRP and KYN were detected on an Agilent 1200 VWD detector (Agilent, Palo Alto, CA, U.S.A.) at 235 nm, KYNA was detected fluorometrically on an Agilent 1260 FLD detector. The acquisition and processing of the chromatograms were performed using an Agilent 1200 system equipped with a Chemstation software (Agilent, Palo Alto, CA, USA). All reagents were p.A. grade from Merck (Darmstadt, Germany). The intra-assay CVs for different concentrations varied between 0.7 - 2.9 % for TRP, 1.7 - 4.3 % for KYN, and 2.6 - 4.5 % for KYNA. The inter-assay CVs for TRP, KYN, and KYNA ranged between 6.3 - 9 %, 2.0 - 5.4 %, and 8.4 - 11.6 %, respectively. 

Creatinine was measured using an enzymatic method applied on a Roche Cobas Mira (Roche Diagnostics, Rotkreuz, Switzerland). The estimated glomerular filtration rate (eGFR) was calculated using the Chronic Kidney Disease Epidemiology Collaboration (CKD-EPI) equation (Levey et al., 2009[16]).

### Beck Depression Inventory (BDI)

The self-reporting 21-question multiple-choice survey (scale: 0 - 3) was used to assess the presence and severity of depressive symptoms. The total values of the BDI range between 0 and 63 points (Beck et al., 1996[4]). According to the literature (Beck et al., 1996[4]; Smarr and Keefer, 2011[26]), subjects with a BDI score > 13 were classified as individuals with the presence of depressive symptoms.

### Statistical analysis

All parameters were recorded in a descriptive statistical manner, tabulated and evaluated. The Kolmogorov-Smirnov test was performed to calculate data distribution. As continuous variables were not normally distributed, they were expressed as medians with interquartile ranges (Q1 - Q3). To calculate potential correlation between two continuous variables the Spearman's rank correlation coefficient (ρ) (not normally distributed data) was used. Univariate linear regression models were performed to assess the association between variables. The Mann-Whitney U test was used for the comparison between two groups. A p value < 0.05 was considered statistically significant. For statistical analysis, the Analyse-it^®^ software version 4.92 (Analyse-it Software, Ltd., Leeds, United Kingdom) was used.

## Results

### Study population characteristics

The baseline demographic and laboratory parameters of the study population are shown in Table 1(Tab. 1). The median age was 39 (range: 18 - 70) years, and 159 (63.6 %) and 91 (36.4 %) were female and male. A total of 114 (45.6 %) individuals were identified with carbohydrate malabsorption. Of these, 50 (20 %) subjects were diagnosed with lactose malabsorption, 49 (19.6 %) with fructose malabsorption, and 15 (6 %) with lactose and fructose malabsorption. The median (Q1 - Q3) BDI score was 10 (3 - 20). In total, 155 (62 %) and 95 (38 %) individuals showed a BDI score ≤ 13 and > 13. 

### Correlations between inflammatory markers with indicators of tryptophan metabolism and signs of depression

Inflammatory markers correlated with indicators of tryptophan metabolism and signs of depression (Table 2(Tab. 2)). Serum IL-6 levels were positively correlated with KYN (p = 0.033, ρ = 0.136), the tryptophan break-down index KYN/TRP (p = 0.005, ρ = 0.176), and with the BDI score (p = 0.001, ρ = 0.205). INF-γ was in tendency positively correlated with KYN (p = 0.058, ρ = 0.120). LBP showed negative correlations with TRP (p = 0.028, ρ = -0.139) and KYNA (p = 0.029, ρ = -0.137), and a positive correlation with the TRP break-down index KYN/TRP (p = 0.005, ρ = 0.177). 

### IL-6, tryptophan metabolism and signs of depression

**Figure 1:**
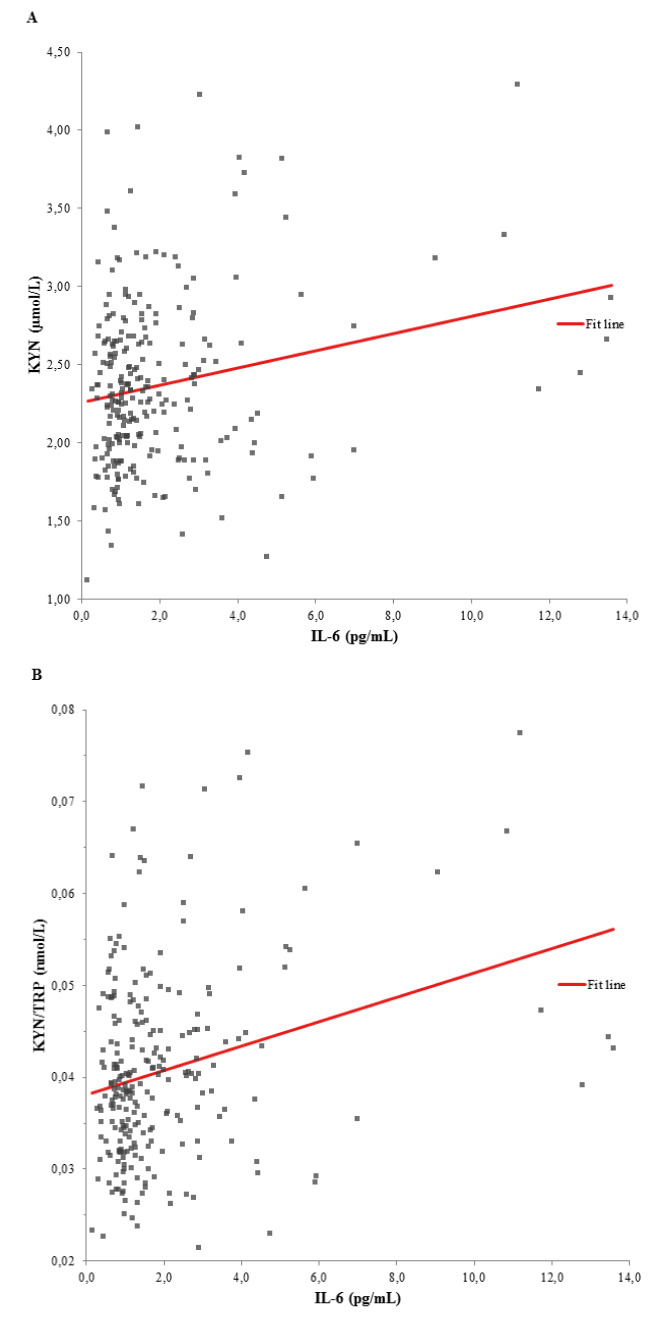
Univariate linear regression models between (A) KYN and IL-6 (ß-coefficient = 0.218, p < 0.001), and (B) KYN/TRP ratio and IL-6 (ß-coefficient = 0.270, p < 0.001). KYN, kynurenine; IL-6, interleukin-6; KYN/TRP, tryptophan break-down index

**Figure 2:**
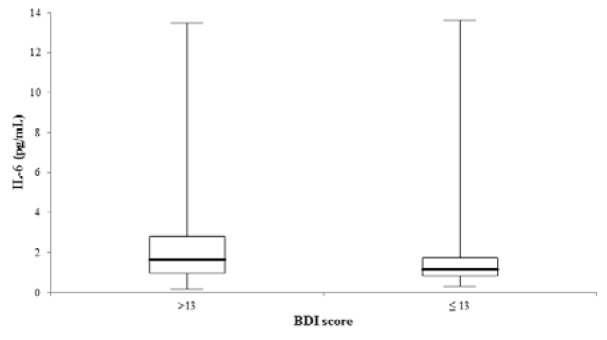
Comparison of IL-6 serum concentrations between 95 and 155 individuals with a BDI score > and ≤ 13 (p < 0.001). The central boxes represent the 25^th^ - 75^th^ percentile range. The lines inside the boxes show the median value for each group. Minimum and maximum are indicated as whiskers with end cups. IL-6, interleukin-6.

As shown in the univariate linear regression models of Figure 1A and 1B(Fig. 1), the tryptophan metabolism indicators KYN (β-coefficient = 0.218, p < 0.001) and KYN/TRP (ß-coefficient = 0.270, p < 0.001) were significantly associated with IL-6. Ninety-five individuals with a BDI score > 13 showed significantly higher IL-6 serum levels (1.7 [1.0 - 2.8] vs. 1.1 [0.8 - 1.7] pg/mL, p < 0.001) compared to 115 individuals with a BDI score ≤ 13 (Figure 2(Fig. 2)). Raw data are provided in Supplementary Table 1.

## Discussion

The present study investigated 250 adult ambulatory subjects referred for carbohydrate malabsorption testing for possible associations between inflammatory markers, TRP metabolism and signs of depression. Serum IL-6 concentrations were positively correlated with KYN and the TRP break-down index (KYN/TRP) and the BDI score. The strongest correlation was observed between IL-6 and the BDI score (ρ = 0.205, p = 0.001).

These findings are in agreement with two previously published studies that found current depressive symptoms measured with the BDI score significantly correlated with IL-6 in patients on maintenance hemodialysis and twins (Hung et al., 2011[12]; Su et al., 2009[28]). In contrast, two recently published reports found no relevant correlation between IL-6 and the severity of depressive symptoms in men with psoriasis and hemodialysis patients (Pietrzak et al., 2018[23]; Knuth et al., 2014[14]). The heterogeneity of study populations and study design variations could be possible explanations of these inconsistent findings. While the present study investigated IL-6 and the BDI score in 250 ambulatory individuals without severe comorbidities, Knuth et al. studied the same parameters in 75 hemodialysis patients, of which 69 (92 %) presented severe chronic comorbidities (92 %) (Knuth et al., 2014[14]). 

Herein, we found a significant association (ß-coefficient = 0.270, p < 0.001) between IL-6 and the KYN/TRP ratio, which was used as indicator of TRP degradation. This result corroborates a previously published study, which reported IL-6 to be associated with the KYN/TRP ratio in 490 chronic dialysis patients (Haverkamp et al., 2017[9]). Recently, inflammation was confirmed as a key in enhancing the TRP break-down along the TRP-KYN pathway in children and adolescents (Michels et al., 2018[18]). IL-6, INF-γ, and TNF-α were shown to accelerate TRP metabolism to produce KYN (Haverkamp et al., 2017[9]; Michels et al., 2018[18]; Fitzgerald et al., 2008[7]; Wang et al., 2020[30]). Present data revealed no relevant correlations between INF-γ and the indicators of TRP metabolism or the severity of depressive symptoms and TNF-α was not detectable in the whole study population. The young mean age and the lack of severe comorbidities in this study cohort might be possible explanations for the current results.

Here, the inflammatory LBP positively correlated with the KYN/TRP ratio (ρ = 0.177, p = 0.005), whereas no relevant correlation was found between lactoferrin and indicators of TRP metabolism. These data indicate, that LBP, which competes with lactoferrin for LPS binding, might also play a key role in the activation of TRP metabolism (Elass-Rochard et al., 1998[5]; Na et al., 2004[21]). A recent study reported significantly increased LBP serum levels in patients with major depressive disorder compared to healthy controls (Alvarez-Mon et al., 2019[2]). Unfortunately, indicators of TRP metabolism were not measured (Alvarez-Mon et al., 2019[2]). LPS is known to induce IDO expression, the key enzyme of TRP catabolism, in an INF-γ-independent way (Wang et al., 2010[31]; O'Connor et al., 2009[22]). Recently published animal models suggest that LPS stimulation induces IL-6 and TNF-α expression with subsequent increasement of the IDO-activity (Garrison et al., 2018[8]; Wirthgen et al., 2014[32]). 

The major limitation of this cross-sectional study is that the authors cannot rule out the influence of antidepressant drugs on the inflammatory status of the patients. However, prospective longitudinal studies with follow-up measurements of inflammatory markers are suggested to get better insight in the associations between inflammation, TRP metabolism and severity of depression.

## Conclusions

The inflammatory markers IL-6 and LBP were shown to be associated with indicators of TRP metabolism. IL-6 was found to be linked to signs of depression. Individuals with the presence of depressive symptoms showed higher serum IL-6 levels compared to individuals without depressive symptoms. These findings corroborate the link between inflammation, TRP metabolism and depression.

## Conflict of interest

The authors declare that there is no conflict of interest.

## Supplementary Material

Supplementary data

## Figures and Tables

**Table 1:**
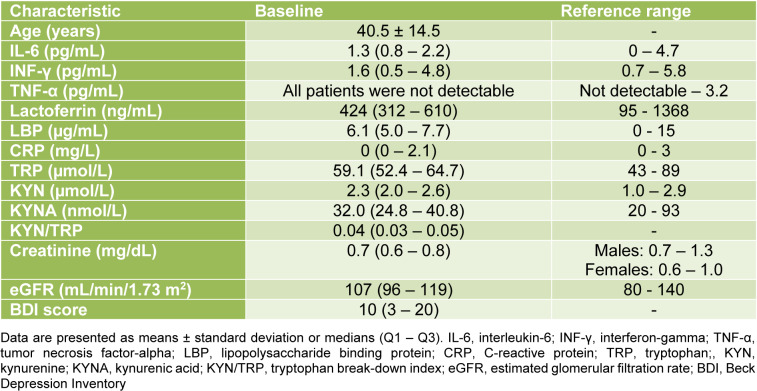
Baseline characteristics of the study population (n = 251)

**Table 2:**
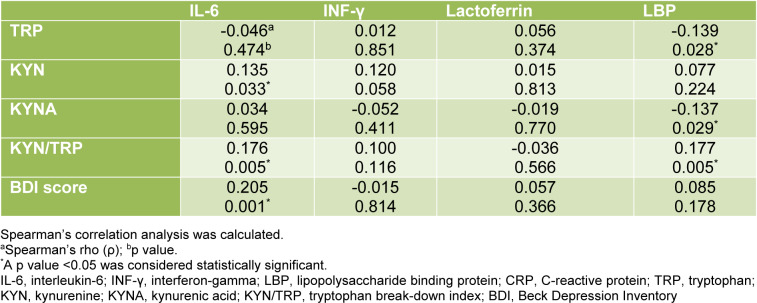
Correlations of IL-6, INF*-γ*, lactoferrin and LBP with indicators of tryptophan metabolism and depressive symptoms
